# COVID-19 Mitigation Plans During Hajj 2020: A Success Story of Zero Cases

**DOI:** 10.1089/hs.2020.0144

**Published:** 2021-04-08

**Authors:** Hani Jokhdar, Anas Khan, Sari Asiri, Wael Motair, Abdullah Assiri, Mohammed Alabdulaali

**Affiliations:** Hani Jokhdar, MBBS, MSc, PhD, is Deputy Minister for Public Health; Anas Khan, MBBS, MHA, SBEM, is Director General, Global Center for Mass Gatherings Medicine; Sari Asiri, MBBS, SBFM, is Director General, Hajj and Umrah Health Services; Wael Motair, MBBS, SBU, is Director General, Health Affairs, Makkah Region; Abdullah Assiri, MBBS, FACP, is Assistant Deputy Minister for Preventive Health; and Mohammed Alabdulaali, is Assistant Minister; all in the Ministry of Health, Riyadh, Saudi Arabia. Anas Khan is also Associate Professor, Emergency and Disaster Medicine, College of Medicine, King Saud University, Riyadh, Saudi Arabia.

**Keywords:** COVID-19, Mass gatherings, Hajj, Public health preparedness/response, National strategy/policy

## Abstract

The Hajj pilgrimage, held in the Kingdom of Saudi Arabia, is among the largest mass gatherings in the world. More than 2.5 million Muslim pilgrims assemble from over 180 countries worldwide to perform Hajj. The Saudi government recognized the potential risks associated with this event since the first novel coronavirus disease 2019 (COVID-19) case was detected in the country on March 2, 2020. The return of possibly infected pilgrims to their countries after this huge mass gathering event could have turned Hajj into a superspreading event during the global COVID-19 pandemic. A multidisciplinary Saudi team from governmental sectors, including the Global Center for Mass Gatherings Medicine, shared in the assessment, planning, execution, and success of this holy event to prevent the spread of disease. The World Health Organization welcomed the Saudi government's decision to protect the wellbeing and safety of pilgrims and strengthen regional and global health security. A total of 1,000 pilgrims from 160 different countries were randomly selected to perform the rituals. Of all the pilgrims, healthcare personnel, and nonmedical employees facilitating the rituals, no confirmed cases of COVID-19 were identified during or after Hajj. This article highlights the success of the risk mitigation plan in place during the Hajj pilgrimage in 2020 (1441 Hijri year) during the COVID-19 pandemic and the efforts of the Saudi government to prevent associated outbreaks.

## Introduction

The world health organization (WHO) declared the novel coronavirus disease 2019 (COVID-19) outbreak a public health emergency of international concern on January 30, 2020. On March 11, 2020, WHO characterized it as a global pandemic.^1^ Since the beginning of this century, our world has experienced several pandemics, but the COVID-19 pandemic has shown unprecedented levels of impact at both national and international levels.^2^ It has had significant repercussions on social, economic, political, and even religious spheres worldwide.^3^

Severe acute respiratory syndrome coronavirus 2 (SARS-CoV-2) is the virus that causes COVID-19 and is well-known for its extremely high transmissibility. This level of transmissibility is a potential threat for proper contact tracing of people who are infected with the disease.^4^ Although the precise definition of *mass gathering* is still up for debate, there is widespread agreement that mass gatherings place a strain on the public health resources of a hosting city or country.^5^ During the COVID-19 pandemic, viral transmission is expected during prolonged or close contact with people who are infected, which makes mass gatherings a serious threat to global health security.^6^ The Kingdom of Saudi Arabia holds one of the largest mass gatherings in human history. Every year, about 2.5 million Muslim pilgrims assemble from over 180 countries worldwide to perform the annual Hajj pilgrimage.^7^ Knowing that infections are common during mass gatherings, respiratory infections could affect up to 80% of pilgrims throughout the 5 days of Hajj.^8^

This article aims to highlight the success story of the 2020 Hajj pilgrimage during the COVID-19 pandemic and the efforts of Saudi authorities in preventing associated outbreaks.

## Saudi Global Center for Mass Gatherings Medicine

The Saudi Ministry of Hajj and Umrah had been aware of the risks associated with the Hajj pilgrimage since the first case of COVID-19 in the country was detected on March 2, 2020.^9^ The government's high level of awareness and readiness resulted from lessons learned during previous pandemics, such as the H1N1 pandemic in 2009.^10^ The Ministry of Health's Global Center for Mass Gatherings Medicine (GCMGM) is a WHO Collaborating Center for Mass Gatherings and showcases the country's ability to develop detection, management, and prevention scenarios for infectious disease outbreaks through years of research.^11^

Since the beginning of the COVID-19 pandemic, WHO has emphasized the significant influence of mass gatherings on economic, political, and social spheres at country levels. WHO encouraged all countries to apply a risk-based approach to properly decide the best scenario for mass gathering events and whether the events should be restricted, modified, postponed, canceled, or held as planned.^12^ Based on WHO recommendations, the GCMGM in Saudi Arabia conducted a health risk assessment for Hajj 2020 (1441 Hijri year). The assessment was conducted using the Jeddah Tool, a health risk assessment framework for mass gatherings, to estimate the health risks of COVID-19 at the pilgrimage. The assessment modeled the expected burden of COVID-19 using severable variables including attack rate, country Hajj quota, and WHO's global disease severity index.^13,14^ The risk assessment identified and prioritized all potential hazards from previous Hajj records and current local and international events and outbreaks using an all-hazard approach. Vulnerability and capacity assessments were then conducted for prioritized hazards to identify the highest risks. The risk assessment also showed that the current number of designated healthcare facilities for the Hajj rituals was found to be severely insufficient to accommodate 2.5 million pilgrims during the COVID-19 pandemic due to high transmissibility.^13,14^

Based on the risk assessment conducted by GCMGM, Saudi authorities decided to reduce the number of pilgrims who could attend the Hajj in 2020. On June 28, 2020, WHO welcomed Saudi Arabia's decision to protect the wellbeing and safety of pilgrims and strengthen regional and global health security.^15^

## COVID-19 National Response Before Hajj Season

Around 75% of the 2.5 million pilgrims who performed Hajj in 2019 were from countries outside the Kingdom, and the majority of pilgrims from within the country were not Saudi nationals. Of the 2.5 million pilgrims, 634,379 were from within the Kingdom and 211,033 of those were Saudi nationals. Saudi nationals represented only about 8.5% of the total pilgrim population. The Saudi Ministry of Health deployed more than 30,000 of its employees to provide healthcare services during Hajj in 2019.^16^ With the unpredictablility of COVID-19 outbreaks, establishing a similar mass gathering in 2020 would have had unanticipated consequences at both national and international levels. Although the Saudi government had applied strict measures to limit the spread of this disease, the numbers of confirmed cases were already on the rise as of April 2020.^9^ By August 15, 2020, almost 26.1 million cases were recorded worldwide and a total of 768,448 deaths from COVID-19 were recorded, with a death rate of 5.1%.^17^ On the same date, 295,902 confirmed cases were reported in Saudi Arabia, with a total of 3,338 deaths related to COVID-19.^18^ Saudi Arabia was one of the countries that took early preventive measures such as suspending international flights and restricting gatherings beginning on March 23, 2020.^19^ The observed low case fatality rate was mainly attributed to extensive testing and a “youth bulge” or high proportion of youths in the population.

Umrah is a smaller Islamic ritual than Hajj and is performed year-round. Umrah involves piligramages to the Great Mosque of Makkah (Al Masjid al Haram) and an optional visit to the Prophet's Mosque of Madinah (Al Masjid an Nabawi), whereas Hajj occurs during specific days each year and involves many rituals including piligrimages to the 2 previously mentioned mosques and other rituals in sacred places such as Mina, Arafat, and Muzdalifa. Umrah was suspended on March 4, 2020, 2 days after the first case of COVID-19 was confirmed, but entrance to Saudi Arabia to perform Umrah was suspended earlier on February 27, 2020.^8^ Prayers were suspended at the 2 mosques in Makkah and Madinah, and Jamaa'h prayers in other mosques around the Kingdom were suspended on March 17. Only employees of the mosques and a group of imams were allowed to enter the mosques, with strict adherence to infection prevention and control measures from the Ministry of Health. These measures had noticeable psychosocial impacts within Saudi Arabia and among Muslims across the globe.^16,20^

**Figure 1.**
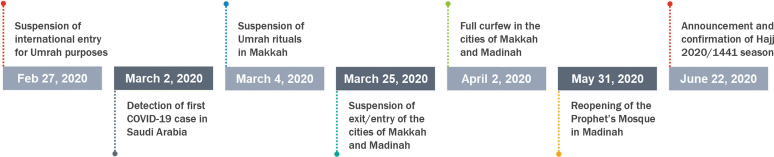
Timeline of Saudi decisions related to Hajj and Umrah.

The series of cancellations and restrictions raised fear and speculation about the cancellation of Hajj in 2020. Prediction models conducted by the Ministry of Health forecasted a significant progression in the number of confirmed cases of COVID-19. In addition to the extremely high risk of having international pilgrims return to their homes with COVID-19, a rising number of cases within Saudi Arabia would have exerted an additional burden on already stretched healthcare systems in Makkah and Madinah. Given the high risks associated with the disease, Alzahrani et al^21^ recommended that Saudi authorities should either cancel Hajj rituals or conduct Hajj with highly restrictive public health measures and mass screening to limit the potential for a massive Hajj-associated surge in cases. The cancellation of Hajj would have had a negative socioeconomic, religious, and political impact in Saudi Arabia and abroad. The Saudi government showed a high level of transparency in its communications as the Ministry of Hajj and Umrah announced that the decision of whether to proceed with the Hajj was evaluated meticulously based on the evolving COVID-19 situation and concerns about the safety of all pilgrims and their home countries. On June 22, in a joint teleconference, the ministers of the Ministry of Hajj and Umrah and the Ministry of Health announced that the Hajj pilgrimage in 2020 would be held with a limited number of pilgrims from within Saudi Arabia and consisting of several nationalities (Figure 1).

## Government Preventive Measures Before Hajj Rituals

The Ministry of Health developed specific eligibility criteria for all candidates who applied to participate in Hajj. Eligibility criteria included that the participant must: (1) be between the ages of 20 and 65 years (with an advisory for candidates older than 50 years), (2) not have certain high-risk chronic diseases, (3) not be obese, (4) not be pregnant, and (5) have a negative polymerase chain reaction (PCR) COVID-19 tests. All eligible candidates were scheduled for appropriate medical examination and screening visits. Each candidate received clear instructions to quarantine for 14 days, including 10 days at home or in a hotel before travel and 4 days in a facility upon arrival in Makkah. Adherence to the quarantine measures was monitored and enforced using a national electronic application known as Tetamman (Arabic for “reassure”). The application monitored daily symptoms and provided educational information for users. It was also linked to a smart wearable electronic tracing bracelet, which generated alerts at the regional Health Command and Control Center if candidates violated quarantine measures. The recommended preventive measures were explicitly stated for all pilgrims. These measures included maintaining a physical distance of approximately 5 feet (1.5 meters) from others, wearing face masks, practicing safe hand hygiene, and disclosing symptoms or contact with a confirmed COVID-19 case in a timely manner. Each candidate completed and signed a written consent document, which detailed their comprehension and willingness to comply with all preventive measures before, during, and after Hajj. They also underwent an assessment of their living conditions to determine its suitability for home quarantine; if deemed unsuitable, they were quarantined in designated hotels. After 10 days of quarantine, all eligible candidates from every region of the Kingdom traveled to Makkah under strict transit measures and were received at another designated facility for the final 4 days of quarantine and repeat PCR testing for COVID-19. Any candidate with a positive PCR test was immediately excluded.

**Figure 2.**
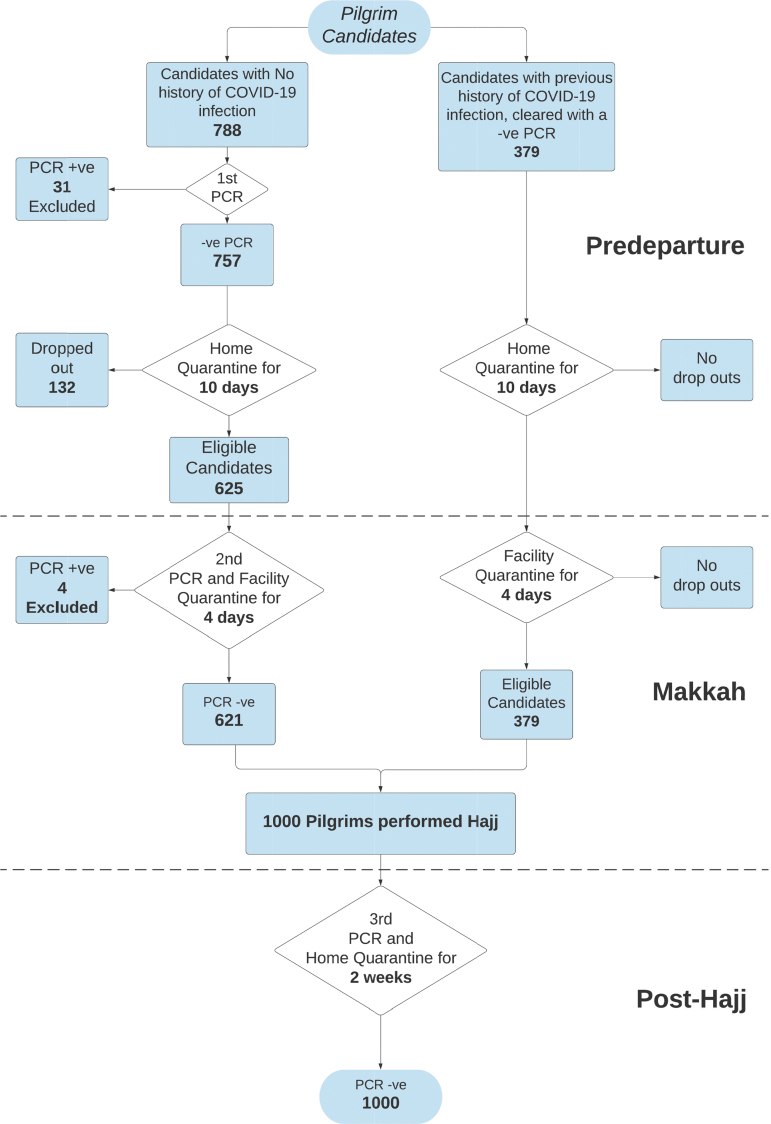
Algorithm and results for PCR testing among all Hajj pilgrim candidates.

**Figure 3.**
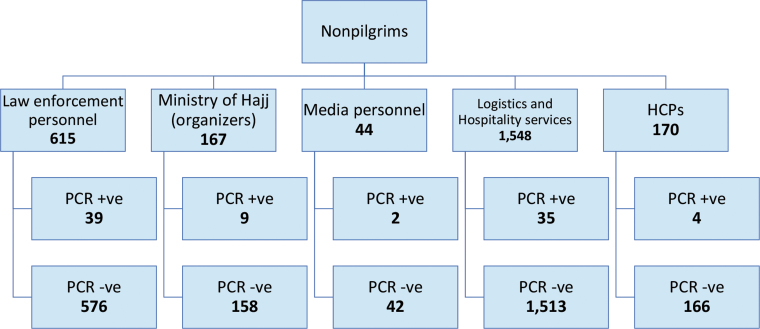
Algorithm and results for PCR testing among nonpilgrims.

Figure 2 presents the results of PCR testing for all pilgrim candidates. During the initial pretravel screening phase, 31 pilgrim candidates tested positive. During the second PCR testing 10 to 14 days later, upon arrival in the Hajj area, 4 additional positive cases were identified. Figure 3 presents the results of PCR testing among the 2,544 nonpilgrims, of whom 89 tested positive. All confirmed COVID-19 cases were transferred to quarantine facilities and received proper medical care. It is unknown why these cases tested positive during the repeat screening, but disease progression and a possible undetected breach of household quarantine measures are potential contributing factors.

## Government Measures During Hajj Rituals

The following measures were planned and implemented during Hajj 2020:

- Safe “bubbles” and tracks – Pilgrims were assigned to groups or “bubbles” of 20 pilgrims, with designated tracks by number and color. These tracks specified, for example, which waiting posts, housing, and bus seats each bubble would use during the long Hajj journey. Group organizers, healthcare professionals, hospitality staff, and drivers were assigned to each group.
- Health officers – A group of 50 trained health officers accompanied the pilgrims during each step of the Hajj rituals to provide the maximum level of care (eg, measuring temperatures frequently, checking symptoms, responding to all medical complaints) and ensure full adherence to preventive measures. Health officers assessed medical concerns accordingly, and arranged expedited transfers to medical posts with dedicated equipment and staff for further management. Standby critical care ambulances were available to transfer patients to designated beds in nearby hospitals if required. The communication and event command system was well designed and applied.
- Hajj community – Special training and eligibility criteria were not just for pilgrims but also included personnel providing services related to Hajj. Personnel were categorized according to their proximity and interaction with pilgrims (ie, hot, warm, or cold zones). Zone passes were provided and measures (eg, PCR testing, quarantine, training) were more stringent for those in hot zones.
- Preventive measures – Measures included the provision of prepackaged meals (no buffets), no sharing of utensils or personal instruments, no physical touching of the Holy Kaaba and other high-touch surfaces, provision of sterile pebbles for each pilgrim for the throwing ritual, and provision of prayer mats for individual use.

The Hajj Health Command and Control Center in Mina was activated, where real-time data on health resources and utilization were closely monitored by decision makers via wide screens and dashboards. Designated healthcare facilities included 2 general hospitals, 4 outpatient clinics, 1 healthcare center in Mina, 1 mobile field hospital, 1 mobile clinic, and 6 ambulance vehicles. A third PCR screening for all pilgrims and personnel was done after performing Hajj rituals and before departure; no confirmed cases were identified.

After finalizing Hajj rituals, all pilgrims continued using the Tetamman application and electronic tracing bracelets to enhance passive surveillance of symptoms (self-reporting) and ensure monitoring of adherence to post-Hajj quarantine measures. In addition to the tracking application, pilgrims received daily phone calls enquiring about their symptoms and monitoring their health status. No cases of COVID-19 were detected.

## Outcomes of the 2020 Hajj Season

**Figure 4.**
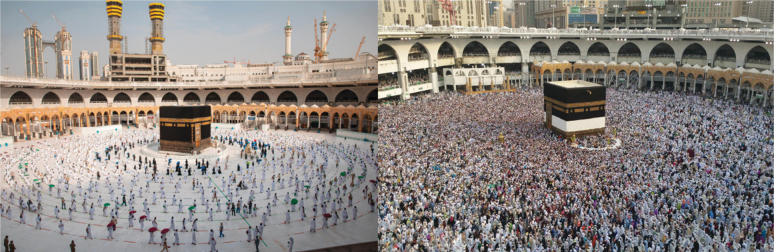
Photos comparing Hajj in 2019 and 2020.

A total of 1,000 pilgrims from 160 different nationalities, living within the borders of the Kingdom, were randomly selected to perform the rituals of Hajj in 2020 (Figure 4). The number of pilgrims included in this year's Hajj was an official decision based on health risk assessment recommendations and political decision making, given the significant socioeconomic, religious, and health security implications. No confirmed cases of COVID-19 were recorded among all pilgrims during or after the end of the holy rituals. Mass gatherings impose grave challenges to healthcare systems and security authorities, especially the COVID-19 pandemic. Reducing the number of pilgrims was a courageous and well-calculated decision based on risk assessment and wide consultation with relevant stakeholders, including religious leaders.^13^ Collaboration between Saudi governmental sectors was one of the critical success factors of the risk mitigation plan. Proceeding with Hajj in 2020 during the COVID-19 pandemic was crucial for several reasons: (1) unlike Umrah, which can be performed anytime, Hajj is an annual event restricted to a specific date in the Hijri lunar year; (2) Hajj has a spiritual and religious place in the hearts of all Muslims worldwide who represent almost 25% of the global population; (3) Hajj has specific and time-limited rituals with a well-known course and order, unlike Umrah; and (4) Hajj is 1 of the 5 pillars of Islam, which makes it an annual destination for millions of Muslims in Saudi Arabia and abroad.

The high risk of importing COVID-19 cases into the Kingdom and exporting cases after Hajj could have led to superspreader events with both national and global implications.^8^ Concerns about Hajj-related international transmission of the disease led to the suspension of Hajj visas by some countries, including Indonesia, before the decision to have a limited Hajj for domestic pilgrims. The exclusion of older people and people with high-risk chronic diseases was also a necessity because they represent higher risks and poorer prognosis and clinical outcomes with COVID-19.^22^ In addition to all precautionary measures and explicit instructions, the exclusion of all confirmed cases before the Hajj rituals was a contributing factor in reaching the final stage of Hajj rituals with no confirmed cases of COVID-19.

## Conclusion

Proceeding with Hajj in 2020 imposed a religious sense of security for the Muslim community worldwide. The decision to proceed with the rituals was based on risk assessments of multiple scenarios in line with WHO guidelines for mass gatherings. The national mitigation plan as outlined in this article aimed to ensure the safety of all pilgrims and personnel and limit the transmission of COVID-19 within and beyond Saudi borders. This model will be replicated and modified for Umrah to ensure the safe return of Umrah as a vital Islamic ritual in the near future. Finally, the success of Hajj in 2020 during the COVID-19 pandemic is a lesson for all global entities. It provides a model for successfully planning, executing, and managing mass gathering events using technology and applying concepts such as safe bubbles, tracks, and assigned health officers, in addition to other proven preventive measures.
